# Valorization of Vegetable Food Waste and By-Products Through Fermentation Processes

**DOI:** 10.3389/fmicb.2020.581997

**Published:** 2020-10-20

**Authors:** Carlos Sabater, Lorena Ruiz, Susana Delgado, Patricia Ruas-Madiedo, Abelardo Margolles

**Affiliations:** ^1^Department of Microbiology and Biochemistry of Dairy Products, Instituto de Productos Lácteos de Asturias, Consejo Superior de Investigaciones Científicas, Villaviciosa, Spain; ^2^Instituto de Investigación Sanitaria del Principado de Asturias, Oviedo, Spain

**Keywords:** food waste, microbial fermentation, by-product Valorization, lactic acid bacteria, bioeconomy

## Abstract

There is a general interest in finding new ways of valorizing fruit and vegetable processing by-products. With this aim, applications of industrial fermentation to improve nutritional value, or to produce biologically active compounds, have been developed. In this sense, the fermentation of a wide variety of by-products including rice, barley, soya, citrus, and milling by-products has been reported. This minireview gives an overview of recent fermentation-based valorization strategies developed in the last 2 years. To aid the designing of new bioprocesses of industrial interest, this minireview also provides a detailed comparison of the fermentation conditions needed to produce specific bioactive compounds through a simple artificial neural network model. Different applications reported have been focused on increasing the nutritional value of vegetable by-products, while several lactic acid bacteria and *Penicillium* species have been used to produce high purity lactic acid. Bacteria and fungi like *Bacillus subtilis, Rhizopus oligosporus*, or *Fusarium flocciferum* may be used to efficiently produce protein extracts with high biological value and a wide variety of functional carbohydrates and glycosidases have been produced employing *Aspergillus, Yarrowia*, and *Trichoderma* species. Fermentative patterns summarized may guide the production of functional ingredients for novel food formulation and the development of low-cost bioprocesses leading to a transition toward a bioeconomy model.

## Introduction

The plant-based food manufacturing sector is currently under expansion and is generating large amounts of by-products. Improved waste management to mitigate the negative environmental impacts of fruit and vegetable processing industries is an essential step in the transition toward a bioeconomy. Therefore, there is a common interest in finding new ways to valorize these substrates, and green valorization schemes that lead to an integrated biorefinery platform have been introduced (Satari and Karimi, 2018). Industrial by-products derived from agriculture, forestry and fishing comprise 1.4% of the total waste production in the European Union (Bedoić et al., 2019). Agricultural waste, co-products and by-products play a relevant role in animal feed production worldwide, while biomass residues are widely used in the production of bioenergy. However, a sustainable bioeconomy must prioritize the production of high-quality foods (Valenti et al., 2020). The generation of these residues strongly depends on land activities, climate conditions, and consumption of goods. It has been reported that Western European countries show a high potential for the use of by-products from vegetable cultivation activities. On the other hand, high volumes of cereal by-products are obtained in Central and Eastern Europe. Valorization of citrus by-products could be of great interest in Southern European countries due to their land areas and mild weather conditions (Bedoić et al., 2019; Valenti et al., 2020), and bioeconomy models for bioenergy generation from Mediterranean feedstocks have been developed (Valenti et al., 2020).

More innovative valorization strategies aim to recover high value ingredients from fruit and vegetable by-products, used as natural sources of biologically active compounds for drug and functional food formulation (Lu et al., 2019). With this aim, fermentative processes using lactic acid bacteria (LAB) and other microorganisms have been described. Fermentative processes can be classified according to different criteria. Most applications developed involve batch fermentations where the substrate and producing microorganism are added to the system at time zero and are not removed until the process is complete. In contrast, in continuous and fed-batch fermentations microorganisms may be immobilized and reutilized for several cycles leading to higher efficiency (Burke et al., 2013). While many industrial fermentations take place in liquid media, in solid-state fermentations microorganisms are grown on a solid support. Shake flasks are the most popular reaction vessels for bioprocessing due to their simplicity, and one-step fermentations have been reported as enhancing mass transfer and reducing potential inhibition by the substrate (Amorim et al., 2019).

Recent examples of fermentation-based valorization strategies include anaerobic digestion of organic feedstocks through mixed culture fermentation (De Groof et al., 2019), fermentation of date palm waste to produce lactic acid as an alternative for the expensive raw material (Azam and Ahmad, 2019), bioconversion of cocoa by-products using bacteria, yeast or filamentous fungi to obtain enzymes, polysaccharides, beverages and nutraceuticals (Vásquez et al., 2019), and sourdough fermentation to elaborate baked foods that may ameliorate the symptoms of irritable bowel syndrome and release bioactive compounds related to the metabolism of phenolic compounds (Gobbetti et al., 2019). In general, most of the applications mentioned above use LAB to improve the nutritional value of several food matrices, or to isolate biologically active ingredients for functional food formulation. Nevertheless, bioconversion processes and fermentation strategies to increase digestibility, enhance nutritional value and decrease the levels of anti-nutritional factors in these substrates using other types of bacteria, as well as different yeasts and molds, have been described (Chebaibi et al., 2019; De Groof et al., 2019; Lücke et al., 2019; Vásquez et al., 2019).

The first part of this minireview provides an overview of recent trends in by-product valorization through microbial fermentation considering: (i) microorganisms selected (LAB, other bacteria, fungi, and yeasts), (ii) main compounds produced and nutritional achievements, (iii) fruit and vegetable wastes and by-products. Then, a comparison of fermentation conditions that should be selected depending on the application as well as a tentative consideration of their economic feasibility are presented.

To select the articles considered in this minireview, we mainly focused on the papers published in the last 2 years and listed in the Web Of Science that were searched using the following keywords and terms: “fermentation,” “vegetable,” and “by-product” or “waste.” Results were then filtered and only research articles reporting fermentation conditions were chosen.

## Fermentation Processes Employing Lab

Lactic acid bacteria (LAB) constitute a group of microorganisms of great industrial interest since they are involved in the production of many fermented foods from raw materials of animal (mainly milk) and vegetable origin (Torres et al., 2020), as well as in feed silage fermentations (Avila and Carvalho, 2020). LAB are highly specialized in the bioconversion of the carbohydrates in lactic acid, rending as well minor amounts of other organic acids which reduce the pH, and are a natural way of conservation; the metabolic activity of LAB on other substrates also have a deep impact in the sensorial, technological, nutritional, and functional characteristics of the resulting fermented foods and feeds (Brückner-Gühmann et al., 2019; Kimoto-Nira et al., 2019; Schettino et al., 2019). The traditional ways of preserving raw materials have favored the “domestication” or selection of specific bacterial lineages well-adapted to the fermented products (Gibbons and Rinker, 2015; Li and Gänzle, 2020). Currently, from this empirical, or non-intentioned, use of LAB comes the application of starter and adjunct cultures from controlled fermentations in the manufacture of a wide variety of fermented foods.

The LAB cultures commonly used in controlled food manufacture are *Streptococcus thermophilus*, *Lactococcus lactis*, *Leuconostoc* spp. and *Lactobacillus* spp., for dairy products, but also the genera *Pediococcus*, *Oenococcus*, and *Weissella* play a pivotal role in plant-based fermented products (Wuyts et al., 2020). Leading approaches are used to improve the characteristics of these industrial bacteria (Hidalgo-Cantabrana et al., 2017; Bron et al., 2019), but the natural resources are also relevant to find novel strains with biotechnological applications (Bachtarzi et al., 2019; Petrova and Petro, 2020). Therefore, the same approaches can be used for the selection of the best LAB candidates to ferment residues from plant materials. Valorization strategies using LAB include the production of lactic acid that may be reintegrated in the food chain as well as enhancing protein digestibility and sensorial properties of these vegetable by-products that could be used as food ingredients (Table 1).

**TABLE 1:** By-product and waste valorization through fermentative processes and enzymatic hydrolysis.

Substrate	Classification	Type of microorganism	Main results	Reintegration in the food chain	References
Rice pasta	By-product	Fungi	Compound production	Yes	Jirasatid et al., 2019
Defatted rice bran	By-product	Bacteria	Compound production	Yes	Alexandri et al., 2019
Rice husk	By-product	Fungi, yeast, and bacteria	Compound production	Yes	Montipó et al., 2019
Rice kernel	By-product	Bacteria	Compound production	Yes	Saman et al., 2019
Rice straw, husk, and bran	By-product	Fungi	Compound production	Yes	Postemsky et al., 2019
Brewer’s spent grain	By-product	Fungi	Compound production	Yes	Amorim et al., 2019
Brewer’s spent grain	By-product	Fungi	Compound production	No	Outeiriño et al., 2019
Brewer’s spent grain	By-product	Fungi	Compound production	Yes	Paz et al., 2019
Brewer’s spent yeast	By-product	Bacteria	Compound production	Yes	Pejin et al., 2019
Brewer’s spent yeast	By-product	Fungi and bacteria	Enhanced properties	Yes	Marson et al., 2019
Barley brans	By-product	Bacteria	Enhanced properties	Yes	Pontonio et al., 2020
Soybean dregs	By-product	Bacteria	Compound production	Yes	Jiang et al., 2019
Soybean meal	By-product	Bacteria	Compound production	Yes	Mukherjee et al., 2019
Soybean meal	By-product	Bacteria	Compound production	Yes	Ruan et al., 2020
Okara (from soymilk)	By-product	Bacteria	Compound production	No	Orts et al., 2019
Soy whey	By-product	Yeast	Compound production	Yes	Chua and Liu, 2020
Soybean cake	By-product	Yeast	Compound production	Yes	Papadaki et al., 2019
Soybean hulls/wheat bran	By-product	Fungi	Compound production	No	Taddia et al., 2019
Wheat bran	By-product	Bacteria	Enhanced properties	Yes	Spaggiari et al., 2020
Pineapple peels	Waste	Fungi	Enhanced properties	Yes	Aruna, 2019
Apple by-products	By-product	Bacteria and yeast	Enhanced properties	Yes	Cantatore et al., 2019
Orange peels	By-product	Bacteria	Compound production	Yes	Ricci et al., 2019b
Mandarin orange waste	Waste	Bacteria	Enhanced properties	No	Tomita et al., 2019
*Citrus depressa* pomace	By-product	Bacteria	Enhanced properties	Yes	Kimoto-Nira et al., 2019
Mango seed	By-product	Fungi	Enhanced properties	Yes	Torres-León et al., 2019
Blueberry pomace	By-product	Bacteria	Enhanced properties	Yes	Cheng et al., 2020
Grape pomace flour	By-product	Fungi	Compound production	Yes	Costa et al., 2019
Melon/Tomato/Carrot	By-product	Bacteria	Enhanced properties	Yes	Ricci et al., 2019a
Fruit and vegetable wastes	Waste	Bacteria	Compound production	No	Yu et al., 2019
Sweet potato distillery by-product	By-product	Fungi	Enhanced properties	Yes	Kosakai et al., 2019
Molasses/potato stillage	By-product	Bacteria	Compound production	Yes	Mladenović et al., 2019a
Molasses/potato stillage	By-product	Bacteria	Compound production	Yes	Mladenović et al., 2019b
Cane molasses	By-product	Yeast	Compound production	Yes	Wang et al., 2019
Maize milling by-products	By-product	Bacteria	Enhanced properties	Yes	Pontonio et al., 2019
Lime cooked maize by-product	By-product	Fungi	Enhanced properties	Yes	Acosta-Estrada et al., 2019
Olive cake	By-product	Fungi	Enhanced properties	Yes	Chebaibi et al., 2019
Olive-mill wastewaters	Waste	Yeast	Compound production	Yes	Sarris et al., 2019
Rapeseed presscake	By-product	Fungi	Enhanced properties	Yes	Lücke et al., 2019
Argan press cake-suspension	Waste	Bacteria	Compound production	Yes	Goto et al., 2019
Hemp/chickpea milling by-products	By-product	Bacteria	Enhanced properties	Yes	Schettino et al., 2019
Grain sorghum flour	By-product	Fungi and bacteria	Enhanced properties	No	Cole et al., 2019
Cassava flour by-product	By-product	Bacteria	Compound production	Yes	do Carmo Brito et al., 2019
Cold-pressing oil products	By-product	Fungi	Compound production	No	Tišma et al., 2019
Oat protein concentrate	By-product	Bacteria	Enhanced properties	Yes	Brückner-Gühmann et al., 2019
Carob pod waste	Waste	Bacteria	Compound production	Yes	Bahry et al., 2019
*Aguamiel* from agave	By-product	Fungi	Compound production	Yes	Muñiz-Márquez et al., 2019

**Species and strains**	**Compounds produced / nutritional achievements**	**Yield / increment (+) / decrease (-)**	**References**		

*Monascus purpureus* TISTR 3541/*Monascus purpureus* TISTR 3629	Pigments	880 OD units/g	Jirasatid et al., 2019		
*Bacillus coagulans* A107	Lactic acid	900 mg/g	Alexandri et al., 2019		
*Penicillium echinulatum* S1M29*/Lactobacillus buchneri* NRRL B-30929*/Saccharomyces cerevisiae* CAT-1	Lactic acid	533 mg/g	Montipó et al., 2019		
*Lactiplantibacillus plantarum* NCIMB 8826*/Limosilactobacillus reuteri* NCIMB 8821	Lactic acid	0.7 g/L	Saman et al., 2019		
*Pleurotus sapidus* MkP6	Nanocellulose	670 mg/g	Postemsky et al., 2019		
*Trichoderma reesei*	Arabinoxylo-oligosaccharides	38 mg/g	Amorim et al., 2019		
*Aspergillus brasiliensis* CECT 2700	Glycosidases	3152 U/g	Outeiriño et al., 2019		
*Aspergillus niger* CECT 2700	Xylose	6 g/L	Paz et al., 2019		
*Lacticaseibacillus rhamnosus* ATCC 7469	Lactic acid	890 mg/g	Pejin et al., 2019		
*Aspergillus oryzae/Bacillus subtilis/Bacillus licheniformis*	Protein content	+50%	Marson et al., 2019		
*Lactiplantibacillus plantarum* T6B10*/Weissella confusa* BAN8	Protein digestibility	+87%	Pontonio et al., 2020		
*Bacillus amyloliquefaciens* NX-2S	Poly-γ-glutamic acid	66 mg/g	Jiang et al., 2019		
*Bacillus coagulans* NCIM 2323*/Lactobacillus johnsonii* LMG 18175	Antioxidant activity	73%	Mukherjee et al., 2019		
*Bacillus subtilis*	Antihypertensive peptides	89 mg/g	Ruan et al., 2020		
*Bacillus licheniformis* ATCC 21415	Glycosidases	2 U/g	Orts et al., 2019		
*Torulaspora delbrueckii*	Organic acids	6 g/L	Chua and Liu, 2020		
*Rhodosporidium toruloides*	Carotenoids	89 μg/g	Papadaki et al., 2019		
*Aspergillus niger* NRRL3	Glycosidases	555 U/mL	Taddia et al., 2019		
*Lacticaseibacillus rhamnosus* 1473	Arabinoxylan solubility	+200%	Spaggiari et al., 2020		
*Trichoderma viride* ATCC 36316	Protein content	+15%	Aruna, 2019		
*Weissella cibaria* PEP23F*/Saccharomyces cerevisiae* AN6Y19	Fiber content	+40%	Cantatore et al., 2019		
*Lacticaseibacillus casei* 2246	Lactic acid	880 mg/g	Ricci et al., 2019a		
*Clostridium beijerinckii* NCIMB8052*/Clostridium cellulovorans* 743B	Sugar content	–85%	Tomita et al., 2019		
*Lactiplantibacillus plantarum* E58	Enhanced sensorial properties	+56%	Kimoto-Nira et al., 2019		
*Aspergillus niger* GH1	Antioxidant activity	+90%	Torres-León et al., 2019		
*Lacticaseibacillus casei*	Phenolic compounds	0.5 mg/mL	Cheng et al., 2020		
*Aspergillus niger* 3T5B8*/Aspergillus aculeatus*	Xylo-oligosaccharides	887 mg/g	Costa et al., 2019		
*Lactiplantibacillus plantarum/Lacticaseibacillus casei/Lacticaseibacillus paracasei/Lacticaseibacillus rhamnosus*	Antimicrobial activity	+700%	Ricci et al., 2019b		
Predominantly *Clostridium kluyveri*	Caproate	620 mg/g	Yu et al., 2019		
*Aspergillus oryzae*	Microbiota modulation: production of SCFAs	+239%	Kosakai et al., 2019		
*Lacticaseibacillus paracasei* NRRL B-4564	Lactic acid	399 g/L	Mladenović et al., 2019a		
*Lacticaseibacillus paracasei* NRRL B-4564	Lactic acid	890 mg/g	Mladenović et al., 2019b		
*Yarrowia lipolytica* S47	Isomaltulose	960 mg/g	Wang et al., 2019		
*Lactiplantibacillus plantarum* T6B10*/Weissella confusa* BAN8	Protein digestibility	+60%	Pontonio et al., 2019		
*Aspergillus oryzae/Pleurotus ostreatus/Hericium erinaceus*	Fiber content	+45%	Acosta-Estrada et al., 2019		
*Aspergillus niger/Beauveria bassiana/Fusarium flocciferum/Rhizodiscina* cf. *lignyota*	Protein content	+94%	Chebaibi et al., 2019		
*Yarrowia lipolytica* ACA-DC 5029	Citric and oleic acid	550 mg/g	Sarris et al., 2019		
*Rhizopus microsporus* var. *oligosporus*	Fiber and protein content	+11%	Lücke et al., 2019		
*Lactiplantibacillus plantarum* Argan-L1	Lactic acid	5 g/L	Goto et al., 2019		
*Lactiplantibacillus plantarum* LB1/*Furfurilactobacillus rossiae* LB5	Fiber and protein content	+13%	Schettino et al., 2019		
*Bacillus licheniformis/Bacillus subtilis/Aspergillus niger/Aspergillus aculeatus*	Increase fermentable sugars	200%	Cole et al., 2019		
*Lactiplantibacillus plantarum/Lactobacillus delbrueckii/Limosilactobacillus fermentum*	Lactic acid	13 g/L	do Carmo Brito et al., 2019		
*Thermomyces lanuginosus*	Lipase	60 U/mL	Tišma et al., 2019		
*Lactobacillus delbrueckii* subsp. *bulgaricus/Streptococcus thermophilus*	Technological properties	+96%	Brückner-Gühmann et al., 2019		
*Lacticaseibacillus rhamnosus*	Lactic acid	22 g/L	Bahry et al., 2019		
*Aspergillus niger* GH1/*Aspergillus niger* PSH*/Aspergillus oryzae* DIA-MF	Fructo-oligosaccharides	470 mg/g	Muñiz-Márquez et al., 2019		

*SCFAs, short-chain fatty acids; U, enzyme activity units; OD, optical density units.*

**FIGURE 1:**
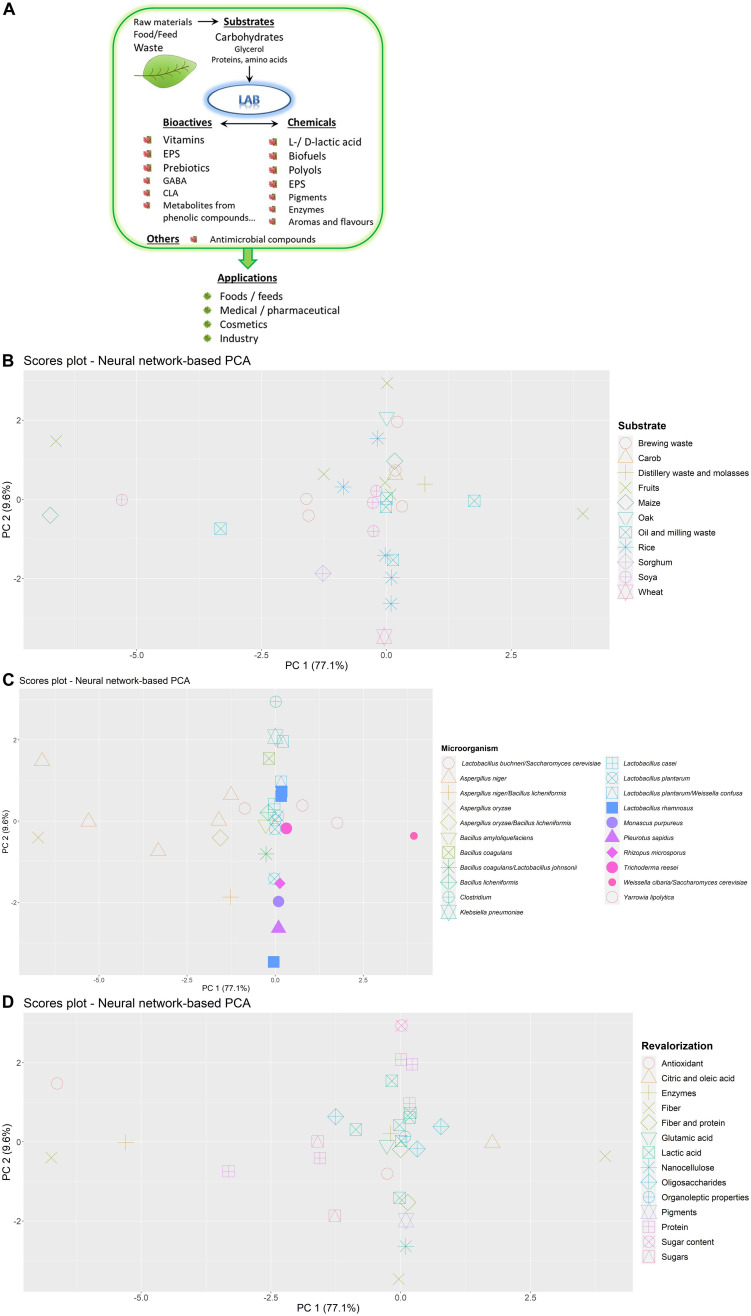
Production of some chemical and/or bioactive compounds from plant-waste by the metabolic activity of lactic acid bacteria (LAB) **(A)** and distribution of fermentation data from studies found in the bibliography by artificial neural network-based principal component analysis (PCA). Differences according to substrate used **(B)**, microorganisms inoculated **(C)** and valorization objective **(D)** and are shown.

As indicated above, the agro-industrial activity generates a wide diversity of waste which is also susceptible to be spontaneously fermented by the microbiota naturally present in these by-products (Verni et al., 2019). Additionally, directed and controlled fermentations can also be driven in order to obtain valorized final products; in spite of the low predominance of LAB in plant autochthonous microbiota, they can also be key players in plant and plant-derived waste fermentations (Filannino et al., 2018). The reason behind this is the high adaptation of LAB to inhabit different plant niches of fruits and vegetables, which include flowers, grains, leaves, and/or grasses (Yu et al., 2020). In addition, Yu et al. (2020) have reported the distinction between generalist- and specialist-LAB in plant ecosystems; the first ones occupy a wide range of habitats but with a variable degree of performance, whilst the specialists are present in a narrow range of habitats and they are highly adapted to them. The generalist-LAB have genomes of bigger size than the specialized once since they harbor a wide enzymatic machinery; an example of the first case is *Lactiplantibacillus plantarum*, widely isolated from plant and animal sources, meanwhile *Fructilactobacillus sanfranciscensis* is mainly associated with cereal (sourdough) fermentations. Another illustrative example of niche specialization is the case of LAB adapted to fructose-rich niches, such as fruits, flowers, their fermented foods, and the intestines of certain insects; these bacteria have evolved to preferably use fructose instead of glucose (Filannino et al., 2019). This group of bacteria can be regarded as cell factories for the production of chemical and bioactives of interest (Figure 1A) due to their metabolic activity upon different substrates (Mazzoli et al., 2014; Sauer et al., 2017; Hatti-Kaul et al., 2018). There is not much information about the ecological distribution of LAB involved in the fermenting of plant waste, although it could be predicted that a similar degree of specialization is expected. Besides, the physical-chemical characteristics of the plant residues are different from those of the raw materials and their fermented food/feed products; thus, it could be expected that generalist LAB, or new specialized species, would be involved in plant-residue fermentations; thus, the waste matrix composition must also be taken into account in order for the selected LAB to optimize their performance during controlled fermentations. There is no doubt, that the fermentation of agro-food residues by LAB, alone or in combination with other microorganisms, opens an avenue of opportunities for a sustainable circular economy.

## Fermentation Processes Employing Other Types of Bacteria

In addition to LAB-mediated fermentations, other fermentative bacteria have been applied to valorize vegetable by-products and other vegetable sources, including different *Clostridium* and *Bacillus* species. Most of these applications focused on the production of functional ingredients like lactic acid, Poly-γ-glutamic acid, bioactive peptides to be reintegrated in the food chain, and other compounds like glycosidases or caproate of industrial interest (Table 1). Within the genus *Bacillus*, *Bacillus coagulans*, *Bacillus amyloliquefaciens, Bacillus licheniformis* and *B. subtilis* have been used, alone or together with other bacteria and/or fungi, to ferment products derived from rice, soy, oak, fruit, sorghum (Alexandri et al., 2019; Cole et al., 2019; Jiang et al., 2019; Mukherjee et al., 2019; Orts et al., 2019). Rice bran is an abundant by-product stream generated during rice processing that, after enzymatic hydrolysis, can be fermented by *B. coagulans* with the aim of producing highly pure (>99%) L-lactic acid. Remarkably, the strain *B. coagulans* A107 was able to convert the sugars of the hydrolyzate to lactic acid with 90% yield, without additional nutritional requirements (Alexandri et al., 2019). On the other hand, soybean dregs, soybean meal and okara can also be fermented by *Bacillus* strains through different valorization ways. Soybean dregs can be transformed using poly-γ-glutamic acid producing stains of *B. amyloliquefaciens* in feed additives, resulting in fermentation products that could help to improve the growth indicators in animal experiments (Jiang et al., 2019). Soybean meal can be fermented by a mixed culture of *B. coagulans* and *Lactobacillus johnsonii*, yielding an improvement of antioxidant properties after fermentation (Mukherjee et al., 2019). Furthermore, *B. licheniformis* was able to ferment enzymatically hydrolyzed okara (a by-product of soy milk manufacturing) to produce soil biostimulants with higher β-glucosidase, phosphatase and dehydrogenase activities (Orts et al., 2019). These three examples show a variety of valorization processes mediated by *Bacillus* species that should be explored further to widen the range of applications of soy-derived products.

Finally, it is worth highlighting the use of *Clostridium* species in the fermentation of fruit waste. In this regard, a successful fermentation strategy using *Clostridium cellulovorans* and *Clostridium beijerinckii* strains was designed to ferment mandarin orange waste. Normally, D-limonene included in citrus fruits inhibits yeast activity and makes ethanolic fermentation difficult. However, physiological concentration of D-limonene does not inhibit the growth of these two *Clostridium* strains. This allows the production of biofuels from this specific fruit waste, thanks to the isopropanol-butanol-ethanol fermentation ability of *C. beijerinckii* and the cellulosic biomass degrading ability of *C. cellulovorans* (Tomita et al., 2019).

## Fermentation Processes Employing Fungi and Yeasts

Fungi probably represent some of the first microorganisms that have been exploited in fermentation processes aimed at producing compounds of interest for medical, nutritional, and industrial applications (Kavanagh, 2005). Fungi produce a diverse array of extracellular enzymes, antibiotics and pigments, and, hence, growing them on vegetable wastes has long been used for their production. Some examples include lactic acid, functional carbohydrates, organic acids or carotenoids production to be reintegrated in the food chain as well as glycosidase and lipase enzymes (Table 1). Applications of fungal fermentation to enhance nutritional properties like fiber and protein content have been also reported (Table 1). For instance, diverse *Aspergillus* species have been used to produce fungal enzymes with important technological applications, such as xylanases through brewer’s spent grain fermentation (Beg et al., 2001); phytases through fermentation of triticale residues (Neira-Vielma et al., 2018), α-amylases through fermentation of soya, wheat bran and other starchy residues (Mathew et al., 2016; Melnichuk et al., 2020), proteases through fermentation of soya bean and wheat bran (Novelli et al., 2015), or lipases through fermentation of lipid-rich agro-wastes such as olive pomaces (Oliveira et al., 2016). Of technological interest is also the production of natural pigments, such as the ones produced by the fermentation of rice pasta with *Monascus purpureus*, a process which also results in the production of an anti-hypercholesterolaemic agent (Jirasatid et al., 2019).

Indeed, fungal fermentation of agri-food wastes is used to produce a number of ingredients with added nutritional and health promoting attributes, either through favoring its release from the vegetable matrix, or through biotransformation of the compounds originally encountered in the residues. Thus, specific compounds produced through these processes are largely dependent on the raw material and the particular fungal species used. For instance, vegetable wastes are a rich source of non-digestible fibers and oligosaccharides which can confer improved technological (Cantatore et al., 2019), nutritional (Chebaibi et al., 2019), and functional properties when included in food preparations. Fungal fermentation of agri-food waste has been frequently used to produce prebiotic substrates, capable of beneficially modulating gut microbial populations (Gibson et al., 2011). In this regard, xylanolytic fungi, such as *Aspergillus* and *Trichoderma* species, produce arabinoxylo-oligosaccharides and xylose from cellulosic rich vegetables wastes, such as brewer’s spent grain, rice husks, soybean hulls or grape pomaces (Amorim et al., 2019; Costa et al., 2019; Paz et al., 2019); and fructo-oligosaccharides have been produced through *Aspergillus* spp. fermentation of *aguamiel* (mead) from agave, sugar cane bagasse, or banana peel and/or leaves (Ganaie et al., 2017; Muñiz-Márquez et al., 2019), whereas isomaltulose, an alternative sweetener with prebiotic properties, is produced through the fermentation of cane molasses (Wang et al., 2019). The capability of generating food supplements with improved functional traits through fungal fermentation has been demonstrated in some works, for instance, supplementing mice diets with a sweet potato distillery waste fermented with *Aspergillus oryzae*, resulted in increased butyrate producers in the gut microbiota and lipid pool modulation (Kosakai et al., 2019).

Fungal fermentation of agri-food residues can also lead to the simultaneous production of multiple ingredients with added value. For instance, fermentation of olive-mill wastewaters by *Yarrowia lipolytica* to produce citric and oleic acid has been reported (Sarris et al., 2019), while a combination of *Aspergillus, Pleurotus*, and *Hericium* enabled the production of fractions rich in polyphenols, antioxidant activities, and fiber from the fermentation of a cooked-maize residue (Acosta-Estrada et al., 2019). Thus, the wide array of metabolic traits exhibited by fungal species offers a valuable scenario to design fermentation strategies capable of maximizing the sustainable production of added-value ingredients through vegetable residue fermentation.

## Comparison of Fermentation Patterns for Different Aplications and Economic Feasibility

Numerous studies dealing with the fermentation of fruit and vegetable by-products as an alternative way of valorization using different microorganisms have been reported in the bibliography. Table 1 provides a classification of substrates assayed in recent works and summarizes cultures and strains selected. Yields of functional ingredients isolated, and enhanced nutritional and technological properties of foods are also shown (reaction conditions for each process are provided in Supplementary Table 1). Moreover, Table 1 compares these fermentative processes which have been carried out in bioreactors to enzymatic hydrolysis using commercial enzyme preparations from bacteria and fungi (Cole et al., 2019; Costa et al., 2019; Marson et al., 2019; Paz et al., 2019).

To find common patterns in fermentation conditions optimized for each type of bioactive compound and nutritional/organoleptic properties, an artificial neural network-based principal component analysis (PCA) is provided in Figures 1B–D. This type of model is especially suitable for data from biological experiments where the relationship between experimental variables is complex, and may help integrating heterogeneous data reported by previous authors to give an overall view of fermentation applications designed for a wide variety of substrates and microorganisms. This model combines a conventional PCA model with artificial neural networks in order to reconstruct experimental data describing as much variance as possible (Stacklies et al., 2007). Artificial neural networks are powerful pattern-recognition algorithms formed by an input layer (i.e., principal components from PCA), an output layer (i.e., reconstructed fermentation conditions) and several neurons or nodes organized in a hidden layer, connected through mathematical functions. In this case, the model was built with six neurons in the hidden layer and was able to explain the high proportion of variance, as shown in Figures 1B–D. Differences and similarities in fermentation processes according to industrial by-products used as substrates (Figure 1B), microbial cultures (Figure 1C), and valorization objectives (Figure 1D) can easily be elucidated through graphical representation.

### Reaction Conditions According to the Substrate

Some differences in fermentative conditions according to each type of fruit and vegetable by-product assayed were observed (Figure 1B). Soya by-products (Jiang et al., 2019; Mukherjee et al., 2019; Orts et al., 2019; Taddia et al., 2019) were mostly subjected to solid-state fermentation at 30–47°C using *Aspergillus niger* and *Bacillus* species or yeasts employing lower temperatures (20–28°C) (Papadaki et al., 2019; Chua and Liu, 2020). Barley bran and brewing waste (Amorim et al., 2019; Marson et al., 2019; Outeiriño et al., 2019; Paz et al., 2019; Pejin et al., 2019; Pontonio et al., 2020) was mostly inoculated with *Aspergillus, Trichoderma*, and LAB species. Rice bran and husk fermentations (Alexandri et al., 2019; Jirasatid et al., 2019; Montipó et al., 2019; Postemsky et al., 2019; Saman et al., 2019) carried out at pH 6.0–6.9 were mainly aimed at lactic acid production compared to other substrates. A great variability was observed in fermentation conditions of fruit by-products depending on the application. Some applications report the use of LAB at different times 72–240 h (Ricci et al., 2019b; Cheng et al., 2020) and no clear patterns could be inferred, although these samples were also differentiated from the rest of substrates.

### Reaction Conditions According to the Microorganism Used

With regard to microbial cultures assayed (Figure 1C), fermentations using *Clostridium*, *B. subtilis*, combination of *B. coagulans* and *L. johnsonii*, *Pleurotus sapidu*s, *A. niger*, and *A. oryzae* showed the highest differences in their reactive conditions. Of all the studies compared, fermentations with mixed cultures of *C. beijerinckii* NCIMB8052 and *C. cellulovorans* 743B to reduce sugar content (−85%) in citrus by-products (Tomita et al., 2019) were carried out with the lowest substrate concentration (1%) at prolonged reaction times (384 h). Similarly, vegetable wastes predominantly fermented by *Clostridium kluyveri* to obtain caproate were carried out at the highest reaction times in a two-step process comprising 80 h and 70 days (Yu et al., 2019). In this sense, fermentations of soya by-products using *B. subtilis* (Ruan et al., 2020) and combination of *B. coagulans* and *L. johnsonii* (Mukherjee et al., 2019) were performed at prolonged times (>140 h) and high initial pH (6.7–7.0). These processes where differentiated from applications using both *Weissella cibaria* PEP23F and *Saccharomyces cerevisiae* AN6Y19 to increase fiber content (+40%) in apple residues (Cantatore et al., 2019) that exhibited low initial pH (4.0), high substrate concentrations and shorter fermentation times (48 h).

On the other hand, some fermentation conditions of rice husks by *P. sapidus* MkP6 to release nanocellulose (670 mg/g) (Postemsky et al., 2019), were similar to those of *Lacticaseibacillus rhamnosus* 1473, to increase arabinoxylan solubility (+200%) from wheat bran (Spaggiari et al., 2020). These two processes were solid-state fermentations where the humidity of substrates was adjusted to 60–75%, with an initial pH of 6.0–6.5. However, these processes were conducted at different times (20 and 48 h). In contrast, *A. oryzae* fermentations of maize-milling waste in co-culture with *Pleurotus ostreatus* and *Hericium erinaceus* to increase fiber content (+45%) (Acosta-Estrada et al., 2019) were performed under similar conditions to those processes using *A. niger*. Specifically, *A. niger* strains NRRL3, GH1 and enzymes isolated from CECT 2700 and 3T5B8 were used to treat mango, grape, soya and brewing by-products, and olive cake, leading to a high antioxidant activity (90%), release of xylo-oligosaccharides (887 mg/g), enzymes (soya 555 U/mL), xylose (6 g/L), and an increase in protein content (+94%) (Chebaibi et al., 2019; Costa et al., 2019; Paz et al., 2019; Taddia et al., 2019; Torres-León et al., 2019). Most of these processes were solid state-fermentations. In general, the use of fungi is preferred to isolate glycosidases and functional oligosaccharides while applications of bacteria are focused on other types of compounds such as lactic acid.

### Reaction Conditions According to the Valorization Objective

Finally, differences in fermentation conditions can also be observed depending on the valorization objective (Figure 1D). Those bioprocesses aimed at releasing oligosaccharides (Amorim et al., 2019; Costa et al., 2019; Muñiz-Márquez et al., 2019; Wang et al., 2019) were characterized by low substrate concentrations (5–35%) inoculated with fungi from *Aspergillus, Trichoderma*, and *Yarrowia* genus at temperatures of 30–40°C. Fermentations designed to produce lactic acid (Alexandri et al., 2019; Bahry et al., 2019; do Carmo Brito et al., 2019; Goto et al., 2019; Mladenović et al., 2019a,b; Montipó et al., 2019; Pejin et al., 2019; Ricci et al., 2019b; Saman et al., 2019) where LAB species were mostly inoculated, were performed with low raw material concentrations (7–25%) at pH 6.0–6.6. Moreover, fermentative applications to increase protein content of matrices (Aruna, 2019; Chebaibi et al., 2019; Marson et al., 2019) were characterized by the use of molds from *Aspergillus* and *Trichoderma* genus. Interestingly, similar reaction conditions were reported to obtain lipases, nanocellulose, and pigments (Jirasatid et al., 2019; Postemsky et al., 2019; Tišma et al., 2019). These processes were solid-state fermentations using fungi at high concentrations of rice and milling by-products (>39%) and an initial pH of 6.0–7.0.

### Economic Feasibility Consideration

Previous studies demonstrate the economic feasibility of vegetable by-product fermentation to obtain high added-value ingredients (Lam et al., 2014; Manandhar and Shah, 2020). Fermentation of vegetable by-products using bacteria, fungi, and yeast showed a return on investment and an internal rate of return above 10%, and a breakeven of the capital investment of approximately 7 years (Lam et al., 2014; Serna-Loaiza et al., 2019; Manandhar and Shah, 2020). To consider the economic feasibility of fermentation applications discussed in this minireview, the selling price for functional ingredients presented in Table 1 was collected and compared to fermentation time and product yield (Supplementary Table 2). It should be noted that only those articles reporting fermentation yields and complete process conditions were considered for comparative purposes. In this sense, the applications that showed the highest ratio between selling price, yield, and fermentation time were the production of xylo-oligosaccharides, poly-γ-glutamic, acid and arabinoxylo-oligosaccharides from grape pomace flour, soybean dregs, and brewer’s spent grain, respectively. This fact is attributed to the high selling price of these ingredients. In contrast, the processes that showed the lowest ratio were the production of carotenoids, isomaltulose and glycosidases from soya by-products and cane molasses, due to the prolonged fermentation times needed and/or lower product price compared to other functional ingredients. It should be noted that no major differences in these ratios were found depending on the microorganism used (bacteria, fungi, or yeast). In summary, production of potentially prebiotic oligosaccharides and biopolymers from vegetable by-products may be highly profitable strategies to valorize vegetable by-products, regardless the microorganism selected. It should be taken into account that the comparison above discussed provides tentative information about the profitability of these applications. However, a detailed cost analysis of each process is needed to further ensure feasibility before their implementation in the industry.

A wide range of bacterial and fungal species to treat several agricultural wastes have been reported in literature. Advances in microbial culture analysis and its integration with omics techniques will boost current understanding of mixed culture fermentation (De Groof et al., 2019) in order to design efficient fermentative processes for specific applications. The present work provides a classification of bacteria and fungi species and strains currently used for bioconversion of fruit and vegetable by-products, and a comparison of fermentative conditions needed to isolate specific functional compounds, or to achieve certain nutritional goals. Most fermentation applications compared in this review could be economically feasible considering the high product yields reported. Among fermentation processes discussed, the most profitable valorization strategies may be the obtainment of functional oligosaccharides and poly-γ-glutamic acid from soya by-products and molasses using *A. niger, A. aculeatus, T. reesei*, or *B. amyloliquefaciens* using specific substrate concentration, temperature, and reaction time conditions. On the other hand, enzyme obtainment from these by-products might not be profitable due to the prolonged fermentation times needed. The information summarized may help in the production of bioactive ingredients for novel food formulation as well as in the development of low-cost bioprocesses leading to a transition toward a bioeconomy model.

## Author Contributions

All authors listed have made a substantial, direct and intellectual contribution to the work, and approved it for publication.

## Conflict of Interest

The authors declare that the research was conducted in the absence of any commercial or financial relationships that could be construed as a potential conflict of interest.
