# Supplementary material for: Valorization of Vegetable Food Waste and By-Products Through Fermentation Processes
Source: Front Microbiol. 2020 Oct 20;11:581997. doi: 10.3389/fmicb.2020.581997 (PMC7606337; doi:10.3389/fmicb.2020.581997)
Supplement: Supplementary file 1 [file Table_1.DOCX]

Supplementary Material

**TABLE S1** **|** Experimental conditions needed for by-product and waste fermentation. **CFU**: colony-forming units, **FPU**: filter paper units.

| **Type of fermentation** | **Substrate concentration (%)** | **Inocula/Dose** | **Time (h)** | **Temperature (°C)** | **Initial pH** | **Final pH** | **Reference** |
| --- | --- | --- | --- | --- | --- | --- | --- |
| Solid-state | 50 | 5% (10^6^ spores/mL) | 336 | 30 | 5.2 - 6.9 | 5.2 - 8.0 | Jirasatid et al. (2019) |
| Batch | 20 | 6% | 30 | 52 | 6.0 | 6.0 | Alexandri et al. (2019) |
| Separate hydrolysis and fermentation | 5 | 20-30 FPU/g | 32+72 | 37 | 5.0 - 6.0 | 5.0 | Montipó et al. (2019) |
| Shake-flask | 3 | 1% (10^7^ CFU/mL) | 30 | 37 | 6.4 - 6.6 | 5.2 - 5.8 | Saman et al. (2019) |
| Solid-state | 39 | 8% | 16-20 | 25 | 6.0 | - | Postemsky et al. (2019) |
| One-step | 20 | 10^6^ spores/mL | 72 | 30 | 7.0 | - | Amorim et al. (2019) |
| Solid-state | 29 | 25 × 10^6^ spores/g | 120 | 30 | - | - | Outeiriño et al. (2019) |
| Enzymatic hydrolysis | 10 | 25 U/mL | 144 | 40 | 5.0 | - | Paz et al. (2019) |
| Batch | 5 | 5% (10^9^ CFU/mL) | 36 | 37 | 6.2 | 3.5 | Pejin et al. (2019) |
| Enzymatic hydrolysis | 100 | 10% | 0.7 | 60 | 5.5 | - | Vollet Marson et al. (2019) |
| Batch | 30 | 10^7^ CFU/g | 24 | 30 | 7.0 | 4.0 - 4.2 | Pontonio et al. (2020) |
| Solid-state | 30 | 10% | 60 | 30 | 8.0 | - | Jiang et al. (2019) |
| Solid-state | 60 | 60% | 144 | 47 | 6.7 | 3.5 - 3.8 | Mukherjee et al. (2019) |
| Liquid (ultrasound-assisted) | 20 | 10% | 168 | 36 | 7.0 | - | Ruan et al. (2020) |
| Batch | 1 | 2% | 312 | 37 | 9.0 | - | Orts et al. (2019) |
| Liquid | 0.35 L | 1% (10^7^ CFU/mL) | 240 | 20 | 4.0 | 3.8 | Chua and Liu (2020) |
| Fed-batch | 7 | 10% | 73-100 | 28 | 5.8 - 6.2 | 5.8 - 6.2 | Papadaki et al. (2019) |
| Solid-state | 20 | 10^6^ conidiospores/mL | 168 | 30 | 6.0 | - | Taddia et al. (2019) |
| Solid-state | 25 | 10^7^ CFU/mL | 48 | 37 | 6.5 | 4.7 | Spaggiari et al. (2020) |
| Solid-state | 40 | 15% (2.7 × 10^6^ spores/mL) | 96 | 30 | - | - | Aruna (2019) |
| Batch | 100 | 4% (10^7^ CFU/g) | 48 | 30 | 4.0 | 3.8 | Cantatore et al. (2019) |
| Solid-state | 25 | 3% (10^7^ CFU/g) | 120 | 37 | 5.0 - 6.5 | 3.8 | Ricci et al. (2019a) |
| Batch | 1 | 5% | 384 | 37 | 5.0 - 7.0 | - | Tomita et al. (2019) |
| Solid-state | 100 | 1% (10^7^ CFU/mL) | 120 | 30 | 4.0 | 3.5 | Kimoto-Nira et al. (2019) |
| Solid-state | 100 | 2 × 10^7^ spores/g | 20 | 30 | 3.7 - 4.8 | - | Torres-León et al. (2019) |
| Batch | 44 | 5% (10^7^ CFU/mL) | 240 | 37 | 3.4 | 3.0 | Cheng et al. (2020) |
| Enzymatic hydrolysis | 5 | 95% | 6 | 40 | 5.0 | - | Costa et al. (2018) |
| Batch | - | 10^7^ CFU/mL | 72 | 30 or 37 | 4.4 - 4.6 | 3.5 | Ricci et al. (2019b) |
| Two-step | 2 | 2:1 inoculum to substrate | 80h and 70 days | 35 and 30 | 6.3 - 7.5 | - | Yu et al. (2019) |
| Liquid | 4 L | 3 x 10^5^ conidiospores/mL | 48 | 30 | - | - | Kosakai et al. (2019) |
| Fed-batch | 16 | 10% inmobilized cells | 5 cycles | 41 | 6.5 | 6.5 | Mladenović et al. (2019a) |
| Fed-batch | 10 | 10% immobilized cells | 3 cycles | 41 | 6.5 | 6.5 | Mladenović et al. (2019b) |
| Fed-batch | 35 | 5% | 80 | 30 | 6.0 | 6.0 | Wang et al. (2019) |
| Batch | 100 | 10^7^ CFU/g | 24 | 30 | 6.2 - 6.4 | 4.0 - 4.2 | Pontonio et al. (2019) |
| Solid-state | 10 | 3% | 72 | 25 | 9.2 | 6.4 | Acosta-Estrada et al. (2019) |
| Solid-state | 67 | 10^6^ spores/mL | 360 | 25 | 5.0 | - | Chebaibi et al. (2019) |
| Shake-flask | 35 | 2% | 242 | 28 | 6.0 | - | Sarris et al. (2019) |
| Batch | 80 | 0.2% | 30-48 | 32 | 5.1 | 5.4 - 8.2 | Lücke et al. (2019) |
| Batch | 5 | 10^8^ CFU/mL | 168 | 37 | 6.4 | 4.0 | Goto et al. (2019) |
| Batch | 63 | 10^7^ CFU/g | 24 | 30 | 6.1 - 6.4 | 4.4 - 4.9 | Schettino et al. (2019) |
| Enzymatic hydrolysis | 18 | 0.001-0.05% | 1.5 | 60 | 5.5 | - | Cole et al. (2019) |
| Spontaneous | 5 L | Spontaneous | 24 | 30 | 6.0 | 4.0 - 4.1 | do Carmo Brito et al. (2019) |
| Solid-state | 60 | 5 mycelial discs (6 mm ⌀) | 216 | 45 | 7.0 | - | Tišma et al. (2019) |
| Batch | 12 | 0.5 - 1 U/L | 24 | 42 | - | 4.1 - 4.2 | Brückner-Gühmann et al. (2019) |
| Batch | 7 | 4% inmobilized cells | 24 | 37 | 6.4 | 6.4 | Bahry et al. (2019) |
| Batch | 0.03 L | 10^7^ spores/mL | 24 | 30 | 4.5 | - | Muñiz-Márquez et al. (2019) |

**TABLE S2** **|** Selling price for different compounds produced from vegetable and fruit by-products and wastes through fermentation and enzymatic hydrolysis. A ratio between selling price, yield and fermentation time (indicated in Table 1 and Table S1) is presented as a tentative indicator of the economic feasibility of the process. Selling price of each product was classified into low (< 1 €/g or €/U), medium (1-100 €/g or €/U) or high (> 100 €/g or €/U). Similarly, the ratio between selling price, yield and fermentation time was classified into low (rate < 1), medium (rate 1-100), high (rate 100-500) or very high (rate >500). **U**: enzyme activity units.

| **Compounds produced** | **Selling price (€/g or €/U)** | **Rate: (Price * Yield)/Time** | **Reference** |
| --- | --- | --- | --- |
| Lactic acid^a^ | Medium | High | Alexandri et al. (2019) |
| Lactic acid^a^ | Medium | Medium | Montipó et al. (2019) |
| Nanocellulose^b^ | Medium | High | Postemsky et al. (2019) |
| Arabinoxylo-oligosaccharides^c^ | High | Very high | Amorim et al. (2019) |
| Lactic acid^a^ | Medium | High | Pejin et al. (2019) |
| Poly-γ-glutamic acid^a^ | High | Very high | Jiang et al. (2019) |
| Antihypertensive peptides^a^ | Medium | Medium | Ruan et al. (2020) |
| Glycosidases^d^ | Low | Low | Orts et al. (2019) |
| Carotenoids^a^ | Medium | Low | Papadaki et al. (2019) |
| Glycosidases^d^ | Low | Low | Taddia et al. (2019) |
| Lactic acid^a^ | Medium | High | Ricci et al. (2019a) |
| Xylo-oligosaccharides^c^ | High | Very high | Costa et al. (2018) |
| Caproate^a^ | High | High | Yu et al. (2019) |
| Isomaltulose^c^ | Low | Low | Wang et al. (2019) |
| Citric and oleic acid^a^ | Medium | Medium | Sarris et al. (2019) |
| Fructo-oligosaccharides^c^ | Medium | Medium | Muñiz-Márquez et al. (2019) |

^a^Estimated selling price taken from Sigma-Aldrich ([www.sigmaaldrich.com](http://www.sigmaaldrich.com)). Selling price for captopril was used as an estimation of the selling price for antihypertensive peptides. Selling price for β-carotene was used as an estimation of the selling price for carotenoids. Selling price for methyl-caproate was used as an estimation of the selling price for caproate.

^b^Estimated selling price taken from Nanografi ([www.nanografi.com](http://www.nanografi.com)).

^c^Estimated selling price taken from Carbosynth ([www.carbosynth.com](http://www.carbosynth.com)). Selling price for xylo-oligosaccharides was used as an estimation of the selling price for arabinoxylo-oligosaccharides.

^d^Estimated selling price taken from Megazyme ([www.megazyme.com](http://www.megazyme.com)). The average selling price for β-glucosidases was used as an estimation of the selling price for glycosidases.
